# Does laparoscopic transperitoneal simple nephrectomy for inflammatory and non-inflammatory kidneys differ?

**DOI:** 10.1186/s40064-016-2945-3

**Published:** 2016-08-17

**Authors:** Omer Kurt, Ibrahim Buldu, Cuneyt Turan, Cenk Murat Yazici

**Affiliations:** 1 Department of Urology, School of Medicine, Namik Kemal University, 59100 Tekirdag, Turkey; 2 Department of Urology, School of Medicine, Mevlana University, Konya, Turkey; 3 Department of Anaesthesiology and Reanimation, School of Medicine, Namik Kemal University, Tekirdag, Turkey; 4 Department of Urology, Ozel Medova Hospital, Konya, Turkey

**Keywords:** Laparoscopy, Nephrectomy, Inflammation, Complication, Transperitoneal

## Abstract

**Background:**

Laparoscopic simple nephrectomy is the standard procedure for the removal of non-functioning benign kidney. It can be performed transperitoneally or retroperitoneally. There are several studies comparing the results of transperitoneally or retroperitoneally laparoscopic nephrectomy but there are limited numbers of study comparing results of laparoscopic transperitoneal simple nephrectomy in non-inflammatory and inflammatory non-functioning kidneys. The aim of this study was to compare the results of laparoscopic transperitoneal simple nephrectomy in non-inflammatory and inflammatory non-functioning kidneys.

**Results:**

We retrospectively reviewed the records of patients who underwent laparoscopic nephrectomy for various inflammatory and non-inflammatory renal conditions at our institution from May 2012 to October 2015. We divided the patients in two groups. Group 2 involved the kidneys with stone disease and/or hydronephrosis, patients with previous renal surgery and patients with the history of recurrent infections. Group 1 involved the patients who had non-functioning kidney without these properties. All the surgeries were performed by transperitoneal approach and peroperative blood loss, operation time, hospitalization time, postoperative creatinine changes and haemoglobin drop were compared between groups. There were 22 patients with inflammatory and 27 patients with non-inflammatory non-functioning kidney. Operation time, peroperative blood loss, hospitalization time, postoperative haemoglobin drop and creatinine difference were not statistically different between groups. Surgical side and the presence of previous surgery did not affect the surgical results of inflammatory and non-inflammatory kidney nephrectomy. The only difference was postoperative fever which was more frequent after the nephrectomy of inflammatory kidney.

**Conclusions:**

On the basis of our experience, surgical results of transperitoneal laparoscopic simple nephrectomy did not differ between inflammatory and non-inflammatory kidneys. Surgical times were higher in inflammatory group even if the difference was not significant.

## Background

Laparoscopic nephrectomy was first performed by Clayman et al. (1991). Since then, this procedure has increasingly been performed. Laparoscopic nephrectomy had many advantages over open technique. Peroperative blood loss, postoperative pain, hospitalization time and patient recovery time were significantly less in laparoscopy cases. Beside these advantages, laparoscopic nephrectomy offered equivalent clinical outcomes. As a result, it became a gold standard technique for the treatment of benign and most of the malignant renal diseases (Fornara et al. 2001; Keeley and Tolley 1998).

Simple nephrectomy is the standard procedure for the removal of non-functioning benign kidney. The term “simple” differentiates the procedure from radical surgery that has been performed for the removal of kidneys with malignancy. Both procedures can be performed by laparoscopy. Simple nephrectomy may be a challenging procedure during laparoscopy. Most of the surgeons dealing with laparoscopic renal surgeries had to deal with fibrotic tissue dissection during simple laparoscopic nephrectomy. Recurrent renal infections, renal stone diseases and previous renal surgeries may cause this fibrotic reaction. In the last decade this challenging conditions were considered as relative contraindication to laparoscopy (Valla 2007).

Comparison between open versus laparoscopic nephrectomy, transperitoneal versus retroperitoneal nephrectomy and laparoscopic versus robotic nephrectomy had been performed in several studies. But there are limited numbers of studies comparing the surgical difficulties and complications of simple laparoscopic nephrectomy in inflammatory and non-inflammatory kidneys. In this study, we compared the results of laparoscopic transperitoneal simple nephrectomy between non-inflammatory and inflammatory non-functioning kidneys.

## Methods

We retrospectively reviewed the records of patients who underwent laparoscopic nephrectomy for various inflammatory and non-inflammatory renal conditions at our institution from May 2012 to October 2015. All patients underwent biochemical and radiological analysis before the surgery. Urinanalysis, urine culture and serum creatinine level were performed as biochemical evaluation. Kidney ureter bladder graph, urinary ultrasonography and non-contrast abdominopelvic tomography were performed as radiological evaluation. Renal functions were evaluated by dimercaptosuccinic asid (DMSA) scintigraphy and the kidneys that had <10 % total renal uptake were defined as non-functioning. Nephrectomy indication was given according to patient’s signs and symptoms. The presence of recurrent urinary tract infection, severe renal pain, unresolved hypertension with multidrug treatment and recurrent macroscopic hematuria constituted the nephrectomy indications for patients.

We divided the patients in two groups. Group 1 involved the patients with non-inflammatory, non-functioning kidneys and group 2 involved the patients with inflammatory non-functioning kidneys. Kidneys with stone disease and/or hydronephrosis, patients with previous renal surgery and patients with the history of recurrent infections were categorized in inflammatory group. Patients who had non-functioning kidney without these properties were categorised in non-inflammatory group. The causes of functional loss of kidneys in non-inflammatory group were hypertension and/or renal arterial diseases. All the surgeries were performed by transperitoneal approach and an informed consent was obtained before the surgery. Peroperative blood loss, operation time, hospitalization time, postoperative creatinine changes and haemoglobin drop were compared between groups.

### Surgical technique

The procedure was performed under general anaesthesia with the patient in full lateral flank position. We used 3 trocars for left nephrectomy and 4 trocars for right nephrectomy. Pnomoperitoneum was constructed at the point, 2 cm cranial to the midline between the umbilicus and superior iliac spine. This site was also used for the placement of first 10 mm trocar. Other trocars were placed under direct vision. Second 10 mm trocar was placed 15 cm cranial to the first trocar and the third 10 mm camera trocar placed medially to the line between the first and the second trocar and laterally to rectus muscle. We used a 5 mm extra trocar for right nephrectomy to retract the liver. We started the dissection with line of Toldt on the right side and medialized the colon and duedonum. The ureter was found and dissected cranially to define the hilar structure of right kidney. The renal vein and artery was identified and doubly clipped and divided. Once the hilar vessels were divided, the dissection continued posteriorly and superiorly to the upper pole, and the adrenal gland was preserved. The ureter was divided and the kidney was removed intact in an EndoBag by extending a 10-mm port. A 20F tube drain was left behind in the surgical space and the trocar sites were closed by 2–0 vicryl and 4–0 prolen suture. Similar surgical procedure was performed in left side. We used 3 trocars for left nephrectomy and the location of trocars were symmetrical to right trocal sites. The colon was medialized and the ureter was found with gonodal vein. Dissection was carried cranially to define the renal vein and renal artery. As the hilar structures were identified, surgery was performed with the same technique that we used for right nephrectomy.

### Statistical analysis

For statistical analysis, SPSS 16.0 version was used. Nonparametric findings were analysed by Chi-square tests. Data in independent groups were analysed for normalcy with Kolmogorov–Smirnov test and further evaluated with independent t-test or Mann–Whitney U test.

## Results

A total of 49 patients were included to study. There were 22 patients with inflammatory and 27 patients with non-inflammatory non-functioning kidney. Demographic properties of study groups are shown in Table 1. Gender, the mean age and the site of surgery were identical between groups. The mean size of inflammatory kidneys was 10.4 ± 3.3 cm. and the mean size of the non-inflammatory kidneys was 6.3 ± 1.3 cm (p < 0.001) The size of the kidneys ranged between 5 and 16 cm. in inflammatory group and 3–8 cm in non-inflammatory group.

**Table 1:** Demographic properties of study groups

	Non-inflammatory kidney	Inflammatory kidney	p value
Female (n)	11	8	p = 0.494
Male (n)	16	14	
Age (mean ± SD)	41.7 ± 17.1	48.9 ± 13.4	p = 0.620
Right kidney (n)	12	10	p = 0.586
Left kidney (n)	15	12	
Size of kidney	6.3 ± 1.3	10.4 ± 3.3	p < 0.001

Operation time, peroperative blood loss, hospitalization time, postoperative haemoglobin drop and creatinine difference were not statistically different between groups. Operation time, blood loss and hospitalization time was higher in inflammatory kidney group but the difference was not significant (Table 2). There was no blood transfusion in both groups and the only complication was postoperative fever, which was significantly higher in inflammatory kidney group. None of the surgeries in both groups were converted to open surgery.

**Table 2:** The relation of peroperative and postoperative variables among groups

	Non inflammatory kidney	Inflammatory kidney	p value
Operation time (min)	117.4 ± 51.7	129.5 ± 45.8	p = 0.372
Blood loss (ml)	72.2 ± 104.4	105.0 ± 133.1	p = 0.219
Hospitalization time (day)	2.3 ± 0.7	3.1 ± 1.0	p = 0.379
Creatinine difference (mg/dl)	+0.10 ± 0.19	+0.07 ± 0.21	p = 0.688
Hemoglobin drop (mg/dl)	1.48 ± 1.25	1.80 ± 1.65	p = 0.297
Postoperative fever (n)	1 (3.7 %)	5 (22.7 %)	p = 0.043

We also evaluated the difference between groups in terms of surgical site and the presence of previous renal surgery. Similar to general results, operation time, blood loss, hospitalization time, creatinine difference and hemoglobin drop did not differ between inflammatory and non-inflammatory group in term of surgical (Table 3). There were 11 patients with the history of previous renal surgery. The mean operation time was 138.6 ± 53.5 min at patients with the history of previous surgery and it was 118.2 ± 47.6 min at patients without previous surgery. Although the operation time was longer in previous surgery group, the difference was not significant (p = 0.706). The mean blood loss, hospitalization time, creatinine difference and hemoglobin drop were also identical between groups.

**Table 3:** Comparison of the variables at inflammatory and non-inflammatory laparoscopic nephrectomies according to the site of surgery

	Right kidney		p value	Left kidney		p value
	Non-inflammatory kidney	Inflammatory kidney		Non-inflammatory kidney	Inflammatory kidney	
Operation time (min)	107.9 ± 51.8	126.0 ± 48.5	0.961	125.0 ± 52.1	132.5 ± 45.3	0.365
Blood loss (ml)	52.5 ± 49.9	58.0 ± 75.0	0.622	88.0 ± 132.9	144.1 ± 159.7	0.439
Hospitalization time (day)	2.2 ± 0.6	2.9 ± 0.9	0.315	2.4 ± 0.9	3.4 ± 1.1	0.604
Creatinine difference (mg/dl)	+0.11 ± 0.17	+0.03 ± 0.12	0.576	+0.09 ± 0.21	+0.10 ± 0.26	0.431
Hemoglobin drop (mg/dl)	1.6 ± 1.5	2.5 ± 1.4	0.669	1.34 ± 1.00	1.18 ± 1.59	0.186

## Discussion

Laparoscopic surgery is the gold standard technique for removal of non-functioning kidney. Patients with laparoscopic surgery had lesser postoperative pain, better cosmetic results, shorter hospital stay and faster recovery time (Rozenberg et al. 1999; Hemal et al. 1999). Laparoscopic nephrectomy can be performed by transperitoneal or retroperitoneal approach. Transperitoneal approach has the advantages of wide surgical field and high manipulation area, which are the major disadvantages of retroperitoneal approach. On the other hand, retroperitoneal approach ensures the surgeons to be away from intraperitoneal organs and led them to reach the renal artery directly, which are the main disadvantages of transperitoneal approach.

The dissection of hilar structures is the major step of both laparoscopic radical and laparoscopic simple nephrectomy. Renal artery must be controlled initially for the following stages. Most of the surgeons dealing with laparoscopic renal surgeries can agree that, laparoscopic simple nephrectomy sometimes can be more challenging than laparoscopic radical nephrectomy. Hilar and perihilar tissue dissection is mostly the main cause of this challenge. Perihilar tissue fibrosis and inflammation makes it hard to identify the hilar structures and to perform adequate dissection. Laparoscopic simple nephrectomy is generally performed for the removal non-functioning kidney without any malignancy. Several pathologies can lead to loss of renal function and most of these pathologies are related with renal and perirenal inflammation. Recurrent renal infections, previous renal surgeries, complicated renal stone diseases, renal tuberculosis and xanthogranulomatous pyelonephritis may cause inflammatory renal function loss. On the other hand, some diseases like systemic hypertension, vascular pathologies and congenital anomalies may result in renal function loss without inflammation. Inflammatory renal conditions have been considered as a relative contraindication for laparoscopic nephrectomy because of higher rates of complications (Bercowsky et al. 1999). Keeley et al reported that the overall complication rate of laparoscopic nephrectomy in their series was 18 %, of which 3 % were major and 15 % were minor complications. The most frequent complication was fever and wound infection. Five (5 %) cases were converted to open surgery and the conversions were especially associated with a history of pyonephrosis, previous renal surgery, staghorn calculi, polycystic kidney disease, and xanthogranulomatous pyelonephritis (Keeley and Tolley 1998). In our study, we did not observe any major complication and we did not convert any surgery to open technique. Conversion rate of laparoscopic simple nephrectomy decreased significantly in the last decade. Technological development and increased experience in laparoscopy might induce the decrease of conversion rates. The introduction of advanced equipment, such as the harmonic scalpel, ligasure and bipolar electric coagulation system, avoided bothersome bleeding and induced easier fibrotic hilar tissue dissection. On the other hand, there was no patient with the diagnosis of xantogranulamatous pyelonephritis, which is the major contraindication for laparoscopic nephrectomy. Postoperative fever was also the most predominant (12.2 %) complication of our patients. It was significantly more frequent in patients with inflammatory kidneys.

There is a controversy in literature concerning the selection of laparoscopic technique for inflammatory non-functioning kidney nephrectomy. Retroperitoneal access enables early control of renal pedicle but it can also be a bothersome technique for the dissection of fibrotic tissue in a limited surgical field. In experienced hands, retroperitoneal laparoscopic simple nephrectomy could be performed successfully with low complication rates. Hemal et al performed retroperitoneal nephrectomy for pyonephrotic nonfunctioning kidneys in 46 patients with a success rate of 88.5 %. Only 6 patients required conversion to open surgery (1 emergently because of colonic injury and 5 electively because of non-progression of the procedure). The mean operation time was 110 min. and the mean blood loss was 95 ml (Hemal et al. 1999). On the other hand, transperitoneal approach is the most frequently preferred technique for the surgery of inflammatory kidney (Siqueira et al. 2002; Rassweiler et al. 1998). Wide surgical field and the familiarity of surgeon to anatomical landmarks makes them feel comfortable for transperitoneal approach. There are several studies demonstrating the surgical results of transperitoneal laparoscopic simple nephrectomy but there are limited numbers of studies comparing the results of transperitoneal laparoscopic simple nephrectomy for inflammatory and non-inflammatory kidneys. Surgeons may think that these reactions complicate the surgery and cause complication. We retrospectively compared the surgical results of laparoscopic nephrectomy in patients with inflammatory and non-inflammatory kidneys. There was no difference between groups in terms of operation time, peroperative blood loss and hospitalization time. The only difference between groups was postoperative fever. Postoperative fever was more frequent after the nephrectomy of inflammatory kidneys. The results were compatible with the literature (Keeley and Tolley 1998; Hemal et al. 1999).

Aminsharifi et al. compared the surgical results of laparoscopic transperitoneal simple nephrectomy between patients who had or had not renal surgery before. The only difference was the operation time. The patients with previous renal surgery had significantly longer operation time. All other parameters like; hospitalization time, blood loss, peroperative and postoperative complications and open conversion rate were similar between groups (Aminsharifi et al. 2011). We also had 11 patients with previous renal surgery. The surgical results including the operation time were similar between the patients with previous surgery and the patients without previous surgery. Laparoscopic nephrectomy in patients with renal stone disease is also often complicated by perirenal adhesions formed secondary to previous episodes of pyelonephritis, pyonephrosis or previous open renal surgery, which makes dissection extremely difficult (Hemal et al. 2001). Soulie et al. (2001) reported that the highest complication rate was in laparoskopik transperitoneal simple nephrectomy group that the renal stone disease was the reason of functional loss. Tepeler et al. (2012) also compared the results of laparoscopic surgery in kidneys with and without stones and they reported that the only difference was operation time. We did not observe any difference between the groups of inflammatory and non-inflammatory kidneys.

We believe that these results were related with the surgical technique that we used for laparoscopic simple nephrectomy. We performed the surgery similar to laparoscopic radical nephrectomy. We did not open Gerota facia untill we dissected adrenal tissue. We opened Gerota facia for dissection of adrenal tissue to leave ipsilateral adrenal in place. We started hilar dissection with the identification of aorta or venacava according to surgical side. The hilar dissection was performed antegradly from the major arteries so we did not have to deal with the perihilar fibrotic tissue. Similar technique was used by some authors before (Hemal et al. 2001; Hemal and Mishra 2010; Duarte et al. 2008). This technique might be useful to perform vascular dissection more easily and quick. The dissection of kidney with perifibrotic tissue can also be performed by subcapsullary. By this surgery, surgeons do not have to deal with perirenal fibrotic tissue but this technique was usually used for retroperitoneal approach (Kapoor et al. 2006).

Our study had some limitations. The small population size and retrospective fashion of our study are the main limitations. We also did not check inflammatory markers before the surgery, which might be an important variable to distinguish inflammatory kidneys form non-inflammatory kidneys. On the other hand, the inflammatory markers that were used in routine exercise may not directly show the inflammatory process in kidneys.

## Conclusions

As a conclusion, surgical results of transperitoneal laparoscopic simple nephrectomy did not differ between inflammatory and non-inflammatory kidneys. The only difference was the rate of postoperative fever, which was more predominant among inflammatory kidneys.
